# Integration of thermocouple microelectrode in the scanning electrochemical microscope at variable temperatures: simultaneous temperature and electrochemical imaging and its kinetic studies

**DOI:** 10.1038/srep43685

**Published:** 2017-03-24

**Authors:** He Pan, Hailing Zhang, Junhui Lai, Xiaoxin Gu, Jianjun Sun, Jing Tang, Tao Jin

**Affiliations:** 1 Ministry of Education & Fujian Provincial Key Laboratory of Analysis and Detection of Food Safety, Department of Chemistry, Fuzhou University, Fuzhou 350116, P.R. China; 2 State Key Laboratory of Physical Chemistry of Solid Surfaces, Department of Chemistry, College of Chemistry and Chemical Engineering, Xiamen University, Xiamen 361005, Fujian, China; 3 College of Electrical Engineering, Fuzhou University, Fuzhou 350116, P.R. China

## Abstract

We describe herein a method for the simultaneous measurement of temperature and electrochemical signal with a new type of thermocouple microelectrode. The thermocouple microelectrode can be used not only as a thermometer but also as a scanning electrochemical microscope (SECM) tip in the reaction between tip-generated bromine and a heated Cu sample. The influence of temperature on the SECM imaging process and the related kinetic parameters have been studied, such as kinetic constant and activation energy.

Temperature is one of the most important parameters in chemical processes^1^, e.g. energy conversion/storage research and biosensor research^2^,^3^,^4^, and is one of the most important quantities in electrochemical reactions^1^,^5^,^6^. Traditional and classical studies in isothermal cells have been performed under isothermal conditions; the whole cell content must be heated (or cooled) either by reaction heat or by external action. Recently, it has been possible to vary temperature as an independent parameter, arbitrarily adjustable like voltage or current. Many methods have been developed for heating electrode surfaces, such as pulse-heated hot wires^7^, screen-printed electrodes on a low-temperature co-fired ceramic base or by applying AC perturbation of low power at extremely high frequencies to microelectrodes^8^,^9^,^10^,^11^,^12^. Different kinds of thermometers have been developed, including the commonly used resistance thermometers^13^, those based on fluorescent molecules^14^, or IR molecular thermometers^15^. However, the lack of high-accuracy reference standards and the specialized and expensive equipment prevent them from becoming generally applied. Previously, a potentiometric method has been devised to visualize the solution temperature gradient at a solid/solution interface using a scanning electrochemical microscope (SECM)^16^. However, this method lacks high accuracy, due to the difficulty in achieving stable readings, which are dependent on the composition of the electrolyte, the pH of the solution, and the solution oxygen concentration.

Therefore, we demonstrate herein *in situ* thermal imaging for the direct detection of the temperature distribution during heating of an electrode by a new type of thermocouple microelectrode^17^. Since the thermocouple microelectrode can be combined with an SECM, this could be developed as a standard and efficient electrochemical technique to investigate heterogeneous and homogeneous reactions in electrolytes. The aim of our work was to develop a method for the simultaneous acquisition of temperature and electrochemical signals that might be used in kinetic studies of electrode reaction. Such a technique with high precision and rapid response could satisfy the requirement to acquire the local temperature image of a substrate during SECM measurement. As a proof of concept, we demonstrate that temperature changes in the vicinity of an electrode can be monitored during electrochemical measurements. The temperature measurements realted with the electrochemical ones could provide more kinetic information about the electrochemical reactions.

## Results and Discussion

**Figure 1:**
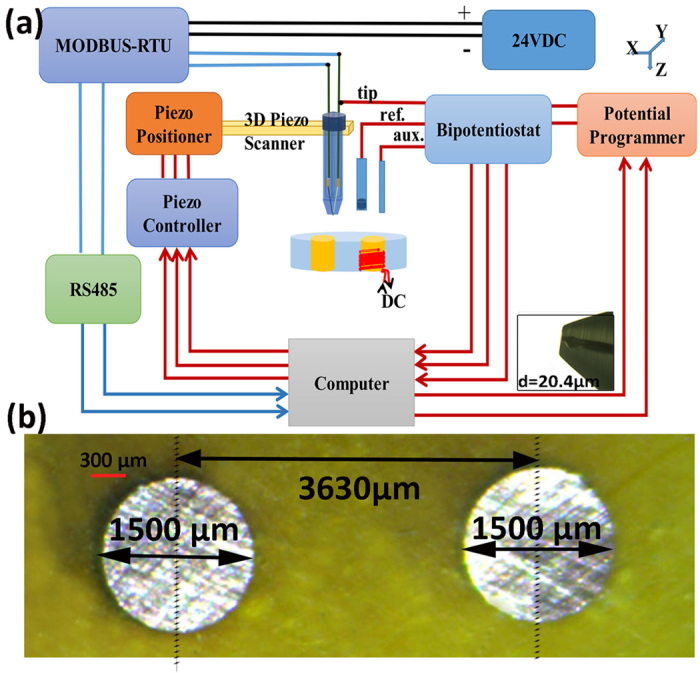
(**a**) Schematic diagram of the integration of an R-type thermocouple temperature detection system and SECM. The inset in (**a**) is a photograph of a Pt–Rh thermocouple microelectrode with a diameter of 20.4 μm. (**b**) Metallurgical microscope image of copper substrate.

**Figure 2:**
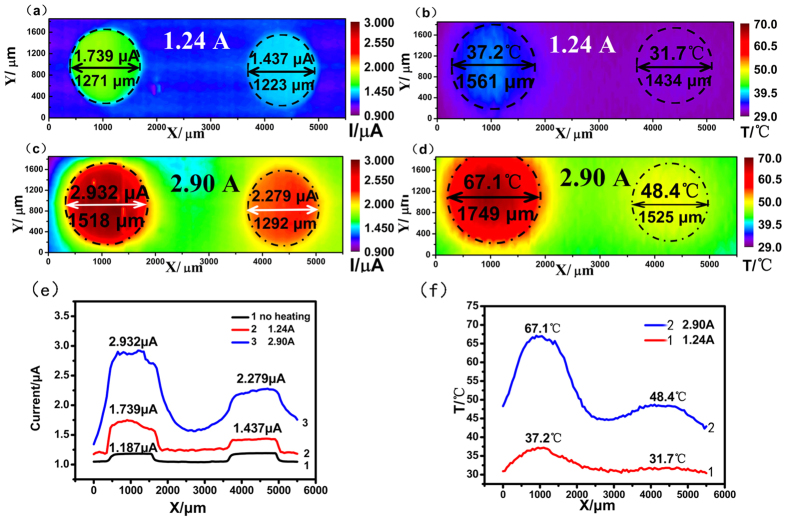
Electrochemical (**a**,**c**) and temperature (**b**,**d**) image of the specially designed Cu substrate in 0.1 M NaBr +2 M H_2_SO_4_ (tip potential = 1.15 V vs. Ag/AgCl; substrate potential = open-circuit potential; tip-to-substrate distance: 20 μm; scan rate: 50 μm·s^−1^, incr. Dist. (μm) = 50, incr. Time(s) = 1; Data collection with MODBUS-RTU temperature acquisition card: one point per second; heating current: 1.24 A (**a**,**b**), 2.90 A (**c**,**d**)). (**e**) Lateral scanning current curves over the copper substrate at different heating current. (**f**) Lateral scanning temperature curves over the copper substrate at different heating current.

Integration of the thermocouple microelectrode with SECM can satisfy the requirements to measure the local temperature around the tip during SECM measurements. In this study, a specially designed substrate adjustable to different temperatures was employed. Two copper electrodes (around 1500 μm in diameter, as shown in Fig. 1(b)) were embedded in a tube, which was then sealed with epoxy resin. One of the two copper electrodes could be heated by means of a double parallel thinner enameled Cu wire wound around it. The temperature of the surface of the electrode was calibrated according to a previous report^18^. The thermocouple microelectrode was calibrated with a Pt100 thermometer in an iced water bath and the resolution was 0.1 °C. The spatial resolution was determined by the size of microelectrode which was 20.4 micrometers in our experiments. A schematic diagram of the experimental set-up is shown in Fig. 1(a). Our previous work has shown that the temperature was increased over room temperature of the electrode and it was linear with the square of the heating current^4^. Figure 2 shows a comparison of the feed back-mode SECM images and temperature images at different temperature from 37.2 °C to 67.1 °C. The temperature was measured by the detection system which consisted the thermocouple microelectrode and temperature acquisition card. The acquisition card could convert the thermoelectric potential and single-chip output to modulus and its frequency, input type, and register address were set according to the sampling points and sampling interval of the SECM imaging. A 24 V DC supply was used to power the temperature acquisition card, and an RS485 adapter was used to connect the temperature acquisition card and computer.

As shown in Fig. 2, the thermocouple microelectrode was operated in the aforementioned solution. Br_2_ was generated at the tip at a potential of 1.15 V vs. Ag/AgCl. The approach curve based on the positive feeback current was employed to position the tip precisely. The distance between the thermocouple tip and the Cu electrode surface was 20 μm and the temperature measured at this distance was proved in our experiments to be the same value as that at the Cu electrode surface. A positive feedback current was observed on the conductive copper surface at the OCP due to the reaction between bromine and the copper substrate and the regenerated bromide ions, whereas a negative feedback current was observed on an insulated bakelite surface^19^,^20^,^21^.

Figure 2(a,c) show SECM images of the Cu substrate, and (b,d) show the simultaneously acquired temperature images. When the DC current was increased from 1.24 A to 2.90 A, the temperature of the copper increased from 37.2 °C to 67.1 °C, as measured by the temperature acquisition system. The measured SECM tip current was increasing from 1.437 μA at 37.2 °C to 2.923 μA at 67.1 °C, as shown in the images in Fig. 2(a,c). Although only one copper wire was heated, the temperature of the other, unheated wire was also increased to 31.7 °C or 48.4 °C due to heat transport across the epoxy resin. The widths of the left copper wire after heating, as shown in the SECM image in Fig. 2(a,c), were 1271 and 1518 μm, respectively. The corresponding diameters of the copper substrate in the images (b) and (d) were 1561 and 1749 μm, respectively. The diameters were measured by the brightest color boundary of the electrochemical and temperature images of the two heated copper wires. It was clearly apparent that the diameters changed simultaneously as the temperature increased. It can be more clearly to compare the temperature influence on the reactions that takes place. When the temperature was measured as 31.7, 37.2, 48.4, and 67.1 °C, the corresponding SECM tip currents were 1.437, 1.739, 2.279, and 2.932 μA, respectively.

The effect of forced convection due to the heated substrate could also be observed from the change in diameter of the temperature field of the copper wires, as measured from Fig. 2(b,d). The diameter of the wire in the temperature images in Fig. 2(b) increased from 1434 μm at 31.7 °C to 1561 μm at37.2 °C. Figure 2(e,f) shows lateral scanning curves of the copper substrate at different heating current, showing that the diameter of the copper electrode in the SECM image and the thermal image increased with increasing temperature, respectively. Figure 2(d) shows that the diameter of the copper electrode increased from 1525 μm at 48.4 °C to 1749 μm at 67.1 °C. The diameter measured from the SECM image was smaller than that from the temperature image. In our experimental investigations, from the SECM image of the Cu substrate in the 0.1 M NaBr + 2 M H_2_SO_4_ solution and the corresponding temperature image, a linear dependence was found between the logarithm of the SECM tip current above the heated Cu sample and the reciprocal temperature under the tip measured by thermcouple microelectrode. (regression coefficient *r* = 0.9747, see Supplementary Fig. S4).

We assume an Arrhenius-type equation (1) of the form:


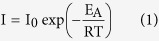


where I is the measured SECM tip current above the heated Cu sample at different temperature T, I_0_ is the pre-exponential factor, and E_A_ is a constant with the dimension of energy which can be calculated as E_A_ = 17.79 kJ·mol^−1^. Cross-sectional analysis of the SECM image and the corresponding temperature image are shown in Fig. 2(e,f).

**Figure 3:**
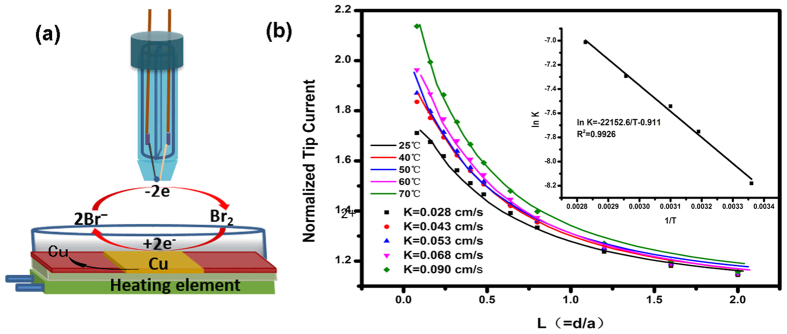
(**a**) Experimental set-up to obtain SECM approach curves on the Cu substrate at different temperatures in 0.1 M NaBr and 2 M H_2_SO_4_. E_tip_ = 1.15 V vs. Ag/AgCl. (**b**) Comparison of the experimental (solid lines) and theoretical (symbols) SECM approach curves. Inset: Arrhenius plot of the simulated kinetic rate of the etching reaction.

This activation energy is ascribed to the total reaction, composed of the electrochemical oxidation of bromide ions and the reaction of bromine with copper, as shown in Fig. 3. The equation of the reaction on the tip above the heated Cu substrate can be decribed as: 2Br^−^ − 2 e → Br_2_ and Br_2_ + Cu → Cu^2+^ + 2Br^−^.

Figure 3(a) shows a schematic diagram of the reaction under the SECM tip. When the SECM tip approaches a surface such as copper, positive feedback is generated since the substrate is reactive with the tip generated bromine. In our experiments, this was caused by the etching reaction, through which Br^−^ ions were regenerated to form a redox loop between the SECM tip and the substrate.

The typical method of measuring the approach curves to study the kinetics of electrode reaction by SECM was employed. After heating the whole SECM cell to different temperatures^22^, approach curves were recorded with a tip potential of 1.15 V vs. Ag/AgCl and a Cu substrate potential of the OCP. It takes only a few seconds to obtain one approach curve, and the etching rate of Cu is relevant to the kinetics of the etching reaction as well as the mass transport of reactants or products in solution.

When the temperature of the copper electrode was increased, the etching reaction rate and the mass transport rate were increased. The finite element modeling method was also used to simulate the tip positive feedback current at different temperatures and the kinetic rate of the etching reaction (*k*) was obtained through COMSOL Multiphysics 4.3b^23^,^24^,^25^. (see Supplementary Fig. S2).

Figure 3 (b) shows that the SECM feedback current curve on the Cu substrate matched the simulated curves at different temperatures. We could thereby obtain the reaction rates (*k*) between bromine and Cu at each temperature: *k* values of 0.028, 0.043, 0.053, 0.068, and 0.090 cm·s^−1^ were obtained at 25, 40, 50, 60, and 70 °C, respectively. (see Supplementary Fig. S3) Once again, we used an Arrhenius plot, equation (2),


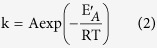


to obtain the reaction activation energy, which according to the etching reaction was calculated as 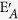
=17.90 kJ·mol^−1^, A is the pre-exponential factor. The simulated profiles of the activation energy by using the obtained values of *k* match the activation results estimated from Fig. S4 very well. The result demonstrated that measuring the electrochemical image and a temperature image simultaneously offers a much faster and more efficient method to acquire information about E_A_. This value 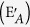 is very close to that measured from the combined temperature and electrochemical images (E_A_), demonstrating that chemical reaction between bromine and the copper substrate played an important role in the total reaction.

**Figure 4:**
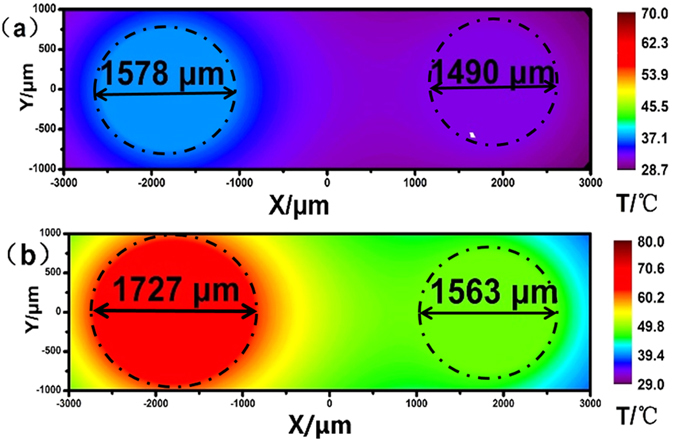
Simulated temperature distributions (**a**,**b**) simulated temperature distributions around the interface of the copper electrode and water at a horizontal cross-section, corresponding to the temperature combinations in Fig. 2(b,d), respectively.

Simulations of the temperature distribution of water around the copper substrate surface were carried out using Fluent software^26^,^27^. A horizontal cross-section with dimensions 6000 μm (horizontal) × 6000 μm (vertical) was simulated. The two copper electrodes (radii 750 μm) were immersed in water at a distance between their centers of 3630 μm. The geometry and meshing were created with the GAMBIT modeling and meshing program. The temperature of the two copper substate was assumed to be the same temperature as shown in Fig. 2(b) and (d). The diameters in Fig. 4(a) were 1490 and 1578 μm which were compared with the Fig.2(b) and the deviations of the theoretical diameters from the measured ones were 3.76% and 1.08%, respectively. The theoretical diameters in Fig. 4(b), corresponding to Fig. 2(d), were 1563 and 1727 μm and the deviations were 2.43% and 1.27%, respectively. A deviation under 4% shows that the temperature measurement by our method achieved high accuracy.

## Conclusions

This new temperature mapping technique offers micrometer spatial and good temperature resolution. Furthermore, it has been integrated with SECM techniques, providing a new, rapid, and accurate methodology to investigate electrochemical and chemical reactions at elevated temperatures. From our initial experimental investigations and COMSOL simulation, the simultaneous measurement of electrochemical information and temperature could be used to calculaete the kinetic constants and the activation energies. These related kinetic parameters could be applied to probe the mechanism of reactions and thereby aid future discoveries concerning heat generation, transfer, and even transport mechanisms during electrochemical reactions in a novel manner.

## Methods

The fabrication procedure for the thermocouple microelectrode has been described in detail in our previous publication^17^. As shown in Fig. 1(a), the temperature detection system consists of a thermocouple microelectrode, a MODBUS-RTU temperature acquisition card and an RS485 adapter (Quanzhou Guanghangda Electronics Technology Corporation, China). The R-type thermocouple temperature detection system is controlled by Kingview software, and was set at 1 s for each point. Excel software was used to store the Kingview data. As shown in Fig. 3(a). A piece of copper (99.999% purity, 1.2 cm × 1.2 cm × 0.1 cm) was used as the substrate to detect the effects of elevated temperature on heterogeneous electron transfer (HET) rates using the thermocouple microelectrode.

The specially designed substrate including two heated Cu wires was prepared as described previously^18^. When a DC power was applied to an enameled Cu wire, heating ensued. The SECM measurement was controlled with a CHI 920C (CH Instruments Inc.) bipotentiostat. When the two copper disc electrodes were not connected to a potentiostat were at the open circuit potential. The reference electrode was Ag/AgCl and a Pt wire served as the counter electrode. In the SECM experiments, the aqueous solution contained 0.1 M NaBr + 2 M H_2_SO_4_.

## Additional Information

**How to cite this article**: Pan, H. *et al*. Integration of thermocouple microelectrode in the scanning electrochemical microscope at variable temperatures: simultaneous temperature and electrochemical imaging and its kinetic studies. *Sci. Rep.* **7**, 43685; doi: 10.1038/srep43685 (2017).

**Publisher's note:** Springer Nature remains neutral with regard to jurisdictional claims in published maps and institutional affiliations.

## Supplementary Material

Supplementary Information
