# Supplementary material for: Integration of thermocouple microelectrode in the scanning electrochemical microscope at variable temperatures: simultaneous temperature and electrochemical imaging and its kinetic studies
Source: Sci Rep. 2017 Mar 24;7:43685. doi: 10.1038/srep43685 (PMC5364503; doi:10.1038/srep43685)
Supplement: Supplementary Information [file srep43685-s1.pdf]

Electronic Supplementary Information

Integration of thermocouple microelectrode in the scanning electrochemical microscope at variable temperatures: simultaneous temperature and electrochemical imaging

He Pan[1], Hailing Zhang[1], Junhui Lai[3], Xiaoxin Gu[2], Jianjun Sun[1], Jing Tang [1,*] and Tao Jin [2,*]

[1] Ministry of Education & Fujian Provincial Key Laboratory of Analysis and Detection of Food Safety, college of Chemistry, Fuzhou University, Fuzhou 350116, PR China
[2]College of Electrical Engineering, Fuzhou University, Fuzhou 350116, P.R. China.
[3]State Key Laboratory of Physical Chemistry of Solid Surfaces, Department of Chemistry, College of Chemistry and Chemical Engineering, Xiamen University, Xiamen 361005, Fujian, China

*** Corresponding authors.**
Telephone and fax numbers: +86 591 22866165
Email: jingtang@fzu.edu.cn; jintly@fzu.edu.cn

Experimental section

Preparation of thermocouple microelectrode

In our experiment， the thermocouple microelectrode was not only used as a thermometer but also employed as an SECM tip in the measurement. The fabrication process of the thermocouple microelectrode is shown in Fig. S1(a) and described as follows. (1) The borosilicate capillary was pulled with a capillary puller to decrease the size of the opening at one end. (2) Pt wire (diameter 25 μm) and 13% Pt–Rh wire (diameter 25 μm) and lengths of almost 1 cm, were connected with two Cu wires using tin solder. (3) The thinning of the wire was achieved by anodic dissolution of the two wire ends in the electrochemical etching solution. A potential of 20 V was applied between the working electrode (Pt or Pt–Rh wire) and a carbon rod counter electrode. (4) The wires were isolated from one another by inserting them into Θ-type quartz capillary. The etched ends of the Pt and PtRh wires weremerged into a spherical joint after melting in a hydroxygen flame. (5) The quartz capillary was inserted into the wide end of the borosilicate capillary and gently pushed to the pulled end. The outer borosilicate capillary, with the spherical joint of Pt and Pt–Rh, was heated by electrical resistance of a Ni–Cr coil and the two kinds of capillaries were sealed together with epoxy resin. (6) The thermocouple microelectrode was polished carefully using emery paper and 0.05 μm alumina suspension before use as an SECM tip. The RG ratio of the diameter of (metal + glass) to the diameter of metal is 3–6.

SECM approach curves were measured in order to accurately determine the RG value of the tip. The thermocouple microelectrode that immersed in 1 mM FcMeOH and 0.1 M KCl solution were moved incrementally toward the conductive surface or insulating surface while biased at a constant potential (0.5 V). The RG could be determined by fitting the experimental approach curves to theoretical approach curves for current over the conductor or insulator.

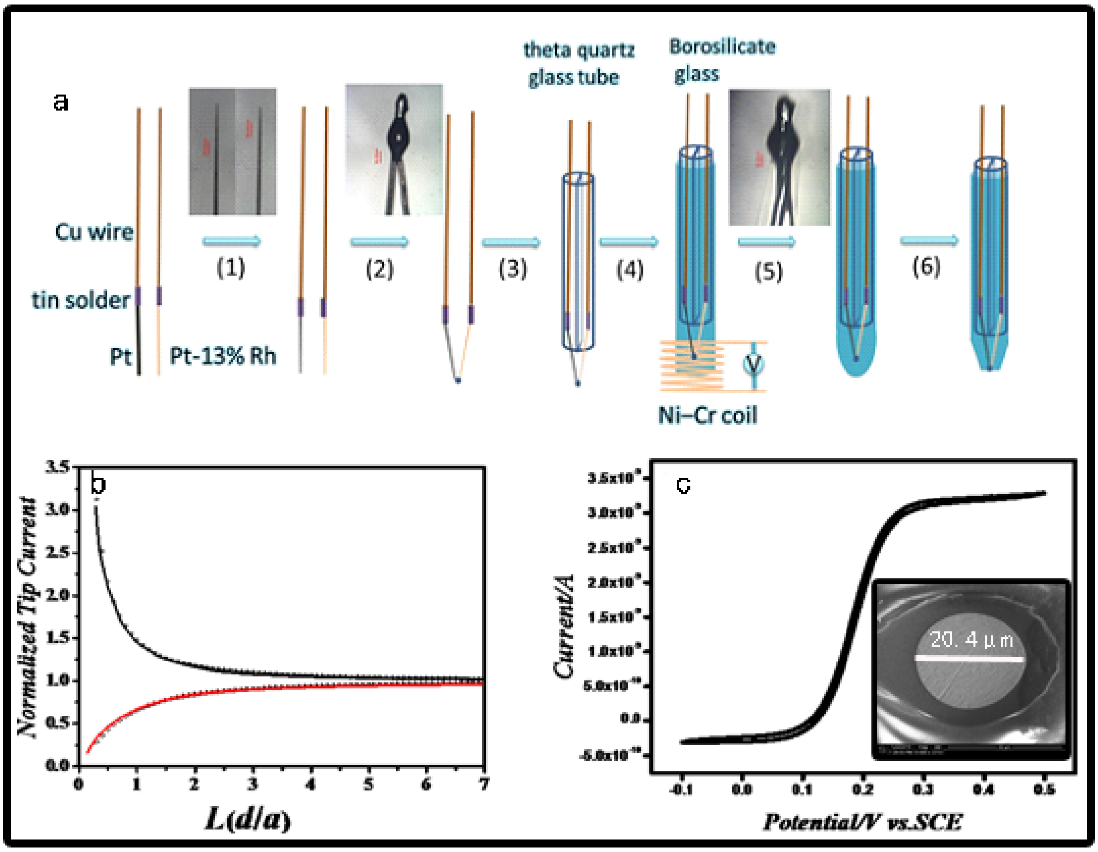

Figure S1. (a) Schematic diagram of the process for preparing a thermocouple micro-electrode. The insets are photos of the corresponding steps. (b) Negative (red solid line) and positive (black solid line) feedback approach curves for a thermocouple microelectrode performed using FcMeOH. E_{tip} = 0.5 V vs. Ag/AgCl. Symbols correspond to theoretical values obtained from an analytical expression. (c) Typical steady-state voltammogram for a thermocouple microelectrode in a 1 mM FcMeOH solution. Potentials are given versus an Ag/AgCl reference electrode. FESEM (inset) of a Pt–Rh thermocouple microelectrode.

An approximate expression for the positive feedback and negative feedback are given by Equation (S1)[2]and (S2)[3]:

$$I_T^c = A + B/L + C\exp(D/L) \tag{S1}$$

$$I_T^{ins} = \frac{1}{[k_1 + k_2/L + k_3\exp(k_4/L)]} \tag{S2}$$

Where I_T^c is the theoretical tip current for the positive feedback and I_T^{ins} is the theoretical tip current for the negative feedback, L is the distance between the tip and the substrate, and A, B, C, D, k_1, k_2, k_3, and k_4 are parameters that change along with the change of RG. When the value of RG is 3, the parameter values are listed in Table S1. As shown in Fig. S1, the experimental approach curves are well fit the theoretical approach curves, so we can confirm the RG of the fabricated

thermocouple microelectrode is 3.0.

Table S1. Parameter values for tip RG=3.

A	*B*	C	*D*	k_1	k_2	k_3	k_4
0.7042	0.7260	0.2751	-1.8699	0.5970	0.8527	0.4035	-1.8567

The radius of the platinum tip (a) was characterized by steady-state voltammetry in a solution of 1 mM FcMeOH and 0.1 M KCl. An approximate analytical expression for tip current at infinite distance from the substrate for a microdisc with finite RG is given by Equation (S3)[4]:

$$i_{T\infty} = 4nFDaC^*\beta_{(RG)} \qquad \text{(S3)}$$

where $i_{T\infty}$ is the limiting current, D is the diffusion coefficient of FcMeOH ($D = 7.8 \times 10^{-6}$ cm^2·s^{-1}), and C^* is the bulk concentration of FcMeOH. $\beta_{(RG)}$ is a correction compared to infinite RG, and the value of $\beta_{(RG)}$ was 1.07 at $RG = 3$. As shown in Fig. S1(b), the limiting current $i_{T\infty}$ was 3.2×10^{-9} A. It was easy to calculate the true radius of the platinum tip using Equation (S3), and the value determined was a = 10.2μm. This thermocouple microelectrode was used in SECM imaging. Thermocouple microelectrodes in our experiment were characterized by the same way.

Finite element method modeling.

Figure S2 shows a schematic diagram of the axisymmetric cylindrical geometry used for the simulation of feedback mode. The numbers in Fig. S2 represent the boundary numbers as defined in Table S2. On the boundaries 1, 4, and 5, there was no flux normal to them for all species. $\vec{n}$ is the inward unit vector normal to the surface. On boundary 2, the oxidation of Br^- on the thermocouple microelectrode tip (RG=5, a=12.5μm) was under diffusion-limited conditions. $C^*_{Br^-}$ is the bulk concentration of the Br^-. On the boundaries 6 and 7, the concentrations of all species are equal to those in the bulk solution. Boundary 3 is representative of the boundary condition of copper substrate, which was defined by flux applying Butler–Volmer kinetics (Equations (S4) and (S5)) for the redox process of Br_2/Br^-.[5]

$$k_b = k^0\exp[-\alpha nf(E - E^{0'})] \qquad \text{(S4)}$$

$$k_f = k^0\exp[(1-\alpha)nf(E - E^{0'})] \qquad \text{(S5)}$$

where k_f is the rate constant for oxidation and k_b is the rate constant for reduction. k^0 is the standard rate constant, α is the transfer coefficient (α=0.5), which is reasonable for the outer-sphere redox process Br_2/Br^-, n is the number of electrons transferred per redox event, and $f = F/RT$, where F is the Faraday constant, R is the gas constant (8.314 J·mol^{-1}·K^{-1}), and T is the temperature. E is the electrode potential and $E^{0'}$ is the formal potential of redox. The tip current was calculated through integrating the flux at the tip surface:

$$i_{tip} = \int_0^a nFD_{Br^-}\frac{\partial C_{Br^-}}{\partial Z}2\pi R dR, n = 1 \qquad \text{(S6)}$$

and the normalized tip current is defined as i_{tip}/i_{ss}, where i_{ss} is the limiting current defined in Equation (S3).

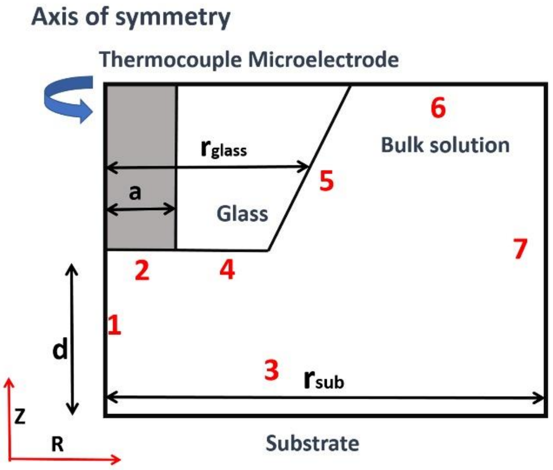

Figure S2. Schematic diagram of the axisymmetric cylindrical geometry used for the simulation of feedback mode.

Table S2. Summary of the boundary conditions used in feedback mode to determine k^0 of different temperature copper substrate.

Label in Fig.S4	Boundary type	Equation
1	Axis of symmetry/insulation	$\nabla C_{Br^-} \cdot \vec{n}=0,\ \nabla C_{Br_2} \cdot \vec{n}=0$
2	Thermocouple microelectrode /concentratio	$C_{Br^-}=0,\ C_{Br_2}=\frac{1}{2}C^*_{Br^-}$
3	copper surface/flux	$-D_{Br_2}\nabla C_{Br_2} \cdot \vec{n} = kC_{Br_2}$<br>$-D_{Br^-}\nabla C_{Br^-} \cdot \vec{n} = -2kC_{Br_2}$
4,5	Glass shear/insulation	$\nabla C_{Br^-} \cdot \vec{n}=0,\ \nabla C_{Br_2} \cdot \vec{n}=0$
6,7	Bulk solution/concentration	$C_{Br^-} = C^*_{Br^-},\ C_{Br_2} = 0$

The measurement of the diffusion coefficient (D) values of Br^- were carried out in a three-electrode cell that immersed in a water bath. A Pt polycrystalline electrode with a diameter of 2 mm was used as the work electrode. The Ag/AgCl electrode was used as the reference electrode and a platinum sheet served as the counter electrode. The concentrations of NaBr and the supporting electrolyte H_2SO_4 were 0.04 M and 2 M, respectively. A series of Cyclic voltammograms curves under different temperature were obtained.

An approximate expression for tip current at different temperature and different diffusion coefficient is given by Equation （S7）:

$$i_p = 0{,}4463[F^3/(RT)]^{1/2}n^{3/2}AD_0^{1/2}C_0^{\ *}v^{1/2} \tag{S7}$$

where i_p is the peak current, D_0 is the diffusion coefficient of Br^-, and C_0* is the bulk concentration of Br^-. A is the electrode area of the Pt polycrystalline electrode . v is the scanning speed， 100 mV/s . The peak current values under the different temperature were shown in Tab. S3. It was easy to calculate the diffusion coefficient (D) values of Br^- using Equation (S7), and the results were displayed in Tab S3.

Table S3. The diffusion coefficient (D) values of Br^- at different temperature.

**Temperature/℃**	**I_p/μA**	**D/((cm^2/s)*10^5)**
**25**	170.2	1.92
**40**	186.9	2.52
**50**	201.1	3.00
**60**	216.5	3.59
**70**	230.7	4.20

Figure S3 shows a series of simulations that were carried out by systematically varying k^0 and d. The corresponding current feedback curves were then obtained with different k^0. The quantitative kinetics of HET on the copper substrate could be obtained by fitting the experimental feedback curves to the simulations. Figure S3 shows that the SECM feedback current curve on the copper substrate matched the simulated curves at different temperatures. We could thereby obtain the reaction rates (k) between bromine and copper at each temperature: k values of 0.028, 0.043, 0.053, 0.068, and 0.090 cm s^{-1} were obtained at 25, 40, 50, 60, and 70 °C, respectively.

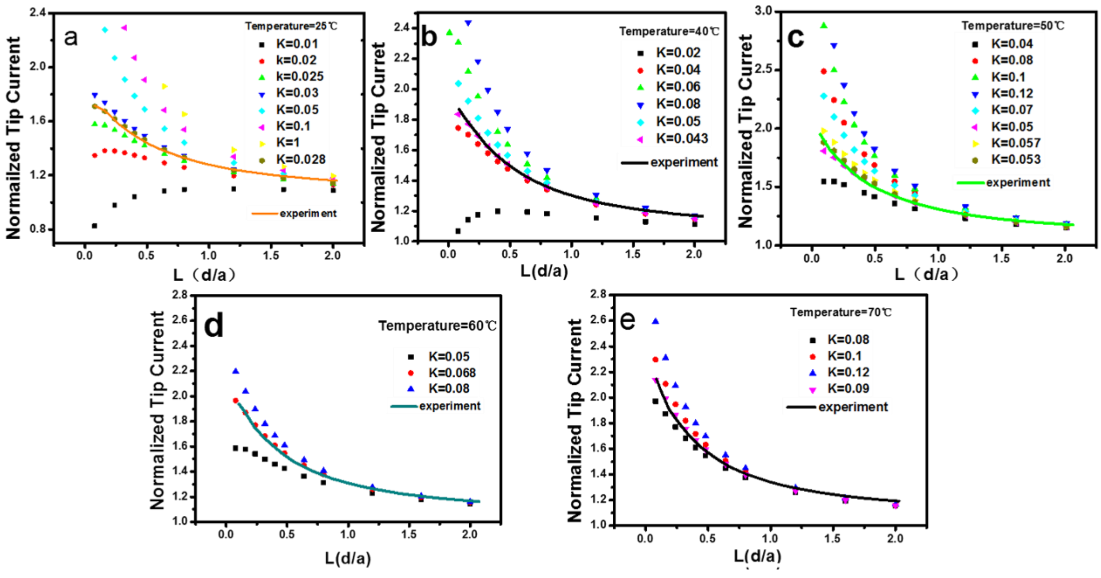

Figure S3. Simulated current feedback curves with different values of k^0. The tip potential was

biased at 1.15 V *vs.* Ag/AgCl (The substrate potential=OCP)

The theoretical simulation by Fluent software

The flow of the solution in our experiment was caused by the substrate heating. So the convection in our experiment belongs to natural convection. According to the equation(S8):

$$Re = \rho VL/\mu \quad (S8)$$

where Re is the Reynolds number, ρ is the fluid density of water (ρ=1000 kg/m3), V is the velocity of flow (V=0.0002 m/s), L is the internal diameter of the pipeline(0.006 m) and μ is the viscosity of liquid(0.001Pa·S). The result shows Re=1.2(<<2300). The range of flow velocity (0.00009 m/s~0.383m/s) was assumed according to the previous literature.[6] Therefore, laminar flow was assumed as the viscous modell. The temperature-dependent viscosity, density, specific heat, and thermal conductivity of water were input to Fluent as data tables using published values.[6,7] The temperature of the substrate surface was 37.2 ℃,31.7 ℃,67.1 ℃,48.4 ℃， respectively. The boundary conditions applied were 25 ℃.

Approaching curve of SECM on the heated copper substrate at different temperatures.

A piece of copper (99.999% purity,1.2 cm*1.2 cm*0.1 cm) was used as the substrate to detect the effects of elevated temperature on heterogeneous electron transfer (HET) rates using the thermocouple microelectrode. When the thermocouple microelectrode was immersed in 0.1 M NaBr and 2 M H_2SO_4 solution, the tip-generated Br_2 can be reduced to bromide ions at the open circuit potential.1 The approach curve based on SECM positive feedback mode was used to position the tip precisely. Then the tip potential was biased at 1.15 V to generate Br_2 and the tip was approached to the copper substrate in order to obtain a series of positive approach curves at different temperatures.

In addition, the copper substrate was heating by our self-made ceramic heating device. The heating apparatus of SECM experiments was similar to that described by Schuhmann, [8] the only difference is that a ceramic heating sheet was used instead of the Peltier element .

The effect of the mapping time

One of the two copper electrodes could be heated by means of a double parallel thinner enameled Cu wire wound around it. The temperature of the surface of the electrode was calibrated according to a previous report.[9] This heating method exhibits both extremely stable and sensitive performance at elevated temperature compared with that at room temperature. Before imaging experiment, the copper electrode would be heated ten minutes

by the direct current. The temperature of the exerperment systerm is stable when the dc voltage remains the same. A duration of 1 s for each point in the temperature image was set by the Kingview software. An increment time of 1 s was also set in the SECM measurement. The scan rate of the SECM tip was 50 μm·s^{-1} (incr. dist. (μm)=50, incr. time (s)=1.), so the mapping time was about 1.4 hours (fig.2 X=5500μm,Y=1800μm). Through repeated experiments, the influence of imaging time could be neglected.

The calculation of activation energy

Figure S4 shows ln(I) plotted against 1/T, the slope of which is a constant value giving E_A=17.79 kJ ·mol^{-1}.

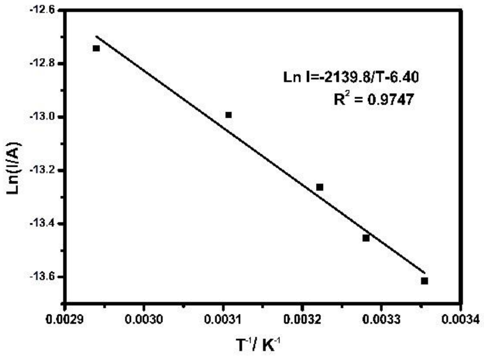

Figure S4. shows ln(I) plotted against 1/T. I is the SECM tip current.

The Relationship between the temperature rise of heating copper electrode and the square of heating current

We used the thermocouple microelectrode to measure the temperature of the copper surface directly. The results (Fig. S5) showed that the temperature increased over room temperature of the electrode is linear with the square of the heating current.

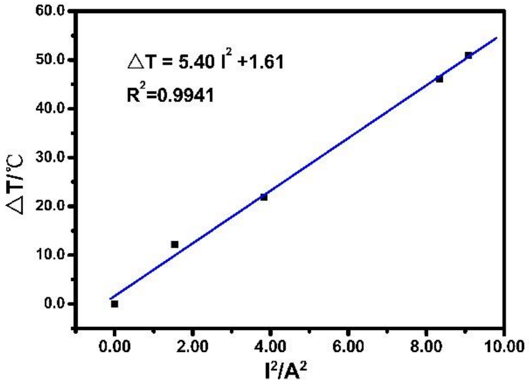

Fig. S5 Relationship between the temperature rise of heating copper electrode and the square of heating current.

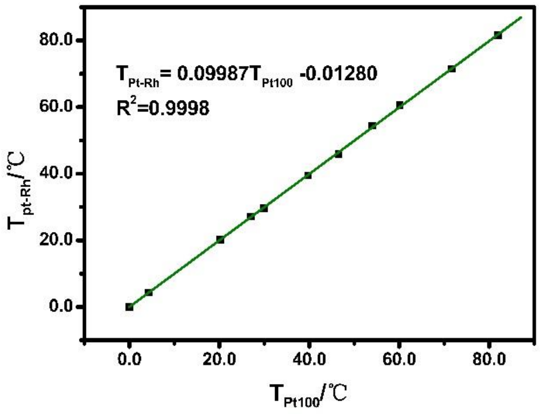

Fig. S6 The calibration of the Pt-PtRh thermocouples by Pt100 thermomete

References

1. Zhang, H., Xiao, X. & Su. T. A novel thermocouple microelectrode for applications in SECM and variable temperature electrochemistry. *Electrochemistry Communications* **47**, 71-74 (2014).
2. Amphlett, J. L. & Denuault, G. Scanning electrochemical microscopy (SECM): An investigation of the effects of tip geometry on amperometric tip response, *J. Phys. Chem. B* **102**, 9946-9951 (1998)
3. Mirkin, M. V. , Fan, F.-R. F. & Bard, A. J. Scanning electrochemical microscopy. Part 13. Evaluation of the tip shapes of nanometer size microelectrodes, *J. Electroanal. Chem.* **328**, 47 - 62 (1992)
4. Lefrou, C & Cornut, R. Analytical Expressions for Quantitative Scanning Electrochemical Microscopy (SECM), ChemPhysChem 11, 547 - 556 (2010)
5. Zhang, J. *et al.* Kinetic Investigation on the Confined Etching System of n-Type Gallium Arsenide by Scanning Electrochemical Microscopy. *J Phys Chem C* **118**, 18604-18611 (2014).

6.Huang, Z. X. et al. Alternate hot and cold electrodes. Electrochemistry Communications 61, 129-133 (2015).

7.Huang, Z. X. et al. Supercooled electrodes. Electrochemistry Communications 48, 107-110 (2014).

8. Schafer, D., Puschhof, A. & Schuhmann, W. Scanning electrochemical microscopy at variable temperatures. *Phys Chem Chem Phys* **15**, 5215-5223 (2013).
9. Wu, S. H. *et al.* Amplified electrochemical hydrogen peroxide reduction based on hemin/G-quadruplex DNAzyme as electrocatalyst at gold particles modified heated copper disk electrode. *Biosens Bioelectron* **73**, 41-46 (2015).
